# Timing and number of antenatal care contacts in low and middle-income countries: Analysis in the Countdown to 2030 priority countries

**DOI:** 10.7189/jogh.10.010502

**Published:** 2020-06

**Authors:** Safia S Jiwani, Agbessi Amouzou-Aguirre, Liliana Carvajal, Doris Chou, Youssouf Keita, Allisyn C Moran, Jennifer Requejo, Sanni Yaya, Lara ME Vaz, Ties Boerma

**Affiliations:** 1 Department of International Health, Johns Hopkins Bloomberg School of Public Health, Baltimore, Maryland, USA; 2 Data and Analytics Section, Division of Data, Analytics, Planning and Monitoring, UNICEF, New York, New York, USA; 3 Department of Reproductive Health and Research, World Health Organization, Geneva, Switzerland; 4 Department of Maternal, Newborn, Child and Adolescent Health and Aging, World Health Organization, Geneva, Switzerland; 5 School of International Development and Global Studies, University of Ottawa, Ottawa, Ontario, Canada; 6 The George Institute for Global Health, The University of Oxford, Oxford, UK; 7 Department of Global Health, Save the Children US, Washington, District of Columbia, USA; 8 Department of Community Health Sciences, Rady Faculty of Health Sciences, Max Rady College of Medicine, University of Manitoba, Winnipeg, Manitoba, Canada

## Abstract

**Background:**

The 2016 World Health Organization (WHO) guidelines for antenatal care (ANC) shift the recommended minimum number of ANC contacts from four to eight, specifying the first contact to occur within the first trimester of pregnancy. We quantify the likelihood of meeting this recommendation in 54 Countdown to 2030 priority countries and identify the characteristics of women being left behind.

**Methods:**

Using 54 Demographic and Health Surveys (DHS) and Multiple Indicator Cluster Surveys (MICS) since 2012, we reported the proportion of women with timely ANC initiation and those who received 8-10 contacts by coverage levels of ANC4+ and by Sustainable Development Goal (SDG) regions. We identified demographic, socio-economic and health systems characteristics of timely ANC initiation and achievement of ANC8+. We ran four multiple regression models to quantify the associations between timing of first ANC and the number and content of ANC received.

**Results:**

Overall, 49.9% of women with ANC1+ and 44.3% of all women had timely ANC initiation; 11.3% achieved ANC8+ and 11.2% received no ANC. Women with timely ANC initiation had 5.2 (95% confidence interval (CI) = 5.0-5.5) and 4.7 (95% CI = 4.4-5.0) times higher odds of receiving four and eight ANC contacts, respectively (*P* < 0.001), and were more likely to receive a higher content of ANC than women with delayed ANC initiation. Regionally, women in Central and Southern Asia had the best performance of timely ANC initiation; Latin America and Caribbean had the highest proportion of women achieving ANC8+. Women who did not initiate ANC in the first trimester or did not achieve 8 contacts were generally poor, single women, with low education, living in rural areas, larger households, having short birth intervals, higher parity, and not giving birth in a health facility nor with a skilled attendant.

**Conclusions:**

Timely ANC initiation is likely to be a major driving force towards meeting the 2016 WHO guidelines for a positive pregnancy experience.

Antenatal care (ANC) is a platform for the delivery of essential services to prevent pregnancy complications, provide counselling for birth and emergency preparedness [1], and improve health outcomes for children [2]. Timely initiation of antenatal care, defined as the first antenatal care contact occurring within the first trimester of pregnancy, provides an opportunity for early screening of modifiable risk-factors and pre-existing conditions [3].

The 2016 WHO recommendations on antenatal care for a positive pregnancy experience suggest a shift from the focused ANC model with a recommended minimum of four ANC visits (ANC4+) to a more expanded model emphasizing number, timing, and content of contacts. This model suggests a minimum of eight ANC contacts, with the first contact taking place in the first trimester of gestation, followed by two and five contacts in the second and third trimesters, respectively [4]. The expansion from a minimum of four to eight ANC contacts highlights the critical need to further target women who initiate ANC late, who are less likely to achieve the new recommended threshold for a positive pregnancy experience.

The achievement of the formerly recommended ANC4+ has been a priority indicator for monitoring maternal health globally and is used in global initiatives such as the Countdown to 2030 [5], the Ending Preventable Maternal Mortality (EPMM) [6], and the Global Strategy for women’s, children’s and adolescents’ health [7]. Conversely, timely first ANC has not been widely reported on, but has been shown to be associated with the total number of ANC contacts achieved and the content of care received [8,9]. Although the global coverage of one skilled ANC contact remains high at 86% [10], a substantially lower proportion of pregnant women initiate ANC during the first trimester [3]. Reasons associated with delayed ANC initiation include financial constraints, distance to health facilities, cultural and religious beliefs around disclosure of pregnancy status, gender norms, lack of awareness of pregnancy signs and antenatal care schedules, pregnancy wantedness, perceptions on the need to start ANC early, and quality of care received [1,11-19].

Nevertheless, little evidence exists on the status of timely ANC initiation on a global scale. A recent study by Moller et al. suggested that the coverage of timely ANC initiation in 2013 was at 24.0% in low-income countries compared to 81.9% in high-income countries [3]. However, further analyses are needed to characterize the profile of women who initiate ANC late, the likelihood of receiving basic components, the standing of countries that are falling behind, and what it will take to improve the timing and number of routine ANC contacts to meet the revised WHO guidelines. Our study addresses these questions in the 81 Countdown to 2030 priority countries [5], accounting for 95% of global maternal deaths and 90% of deaths among children under-five globally. Specifically, we aim to (1) quantify the timing of ANC initiation and its variation according to overall levels of ANC contacts, (2) compare the profile of women who initiated ANC during the first trimester to that of women who delayed the first visit until the second or third trimester, (3) quantify the association between timely ANC initiation and a) the number of ANC contacts received and b) the content of ANC care received, (4) estimate the proportion of women who achieve ANC8+ contacts and compare their profile to that of women with 1-7 ANC contacts.

## METHODS

### Data

We searched the latest available Demographic and Health Surveys (DHS) and Multiple Indicator Cluster Surveys (MICS) of all 81 Countdown to 2030 priority countries since 2012 for data on timing of first ANC contact and number of ANC contacts achieved. The year 2012 cut off was used to ensure that estimates produced are recent across the countries included in the analysis. 54 countries with latest DHS or MICS surveys carried out between 2012 and May 2018 were included in the analysis: 45 DHS and 9 MICS surveys. We restricted the analysis to the last live birth in the two years preceding the survey reported by women aged 15-49 interviewed during the survey. The 54 surveys include a total of 290 783 such births, ranging from 932 in Guyana to 91 614 in India, with available data on timing and number of ANC (Table S1 in the **Online Supplementary Document**).

The DHS and MICS are nationally-representative household surveys implemented in over 90 low- and middle-income countries that provide data on health and population indicators. The surveys include samples of women of reproductive age, generally 15-49 years, who are asked about information on their pregnancies in the last five years or their last pregnancy. Both sets of surveys collect information on ANC, including the timing of first ANC contact through the question “how many weeks/months pregnant were you when you first received antenatal care for this pregnancy?”. Information on the total number of ANC contacts and selected basic ANC components received are also collected.

### Main variables

Our main outcome of interest, timing of antenatal care, was the self-reported gestational age at which the respondent had her first ANC contact during her pregnancy for the index child. We recoded the gestational age variable collected in DHS and MICS into months from one to nine and defined timely initiation of ANC as a first contact occurring within the first trimester. Additional variables included the total number of ANC contacts received and the content of care received, both captured as self-reported variables in DHS and MICS. The number of ANC contacts was further grouped into ANC4+ and ANC8+.

To assess receipt of basic ANC components during ANC contacts, we relied on the limited information collected in the surveys on content of care received at least once during ANC, available across both type of surveys. These include blood pressure measurement, urine test, blood test, and HIV testing and receipt of result. Although they do not capture the full set of components required during ANC, together they provide an indication of whether some basic services could be recalled by the women. We created a content of antenatal care variable as a categorical score (0, 1, 2+), defined as the number of components reported to have been received at least once during pregnancy, out of the total of four components considered.

### Statistical analysis

We carried out statistical analyses using individual country data sets as well as a pooled data set of all countries. For the latter, the analysis was weighted by the inverse proportion of sampled births contributed by each country in the pooled data set, to adjust for the size of the sample of each country data set. We analyzed the timing of ANC for the total sample as well as by three groups of countries based on their coverage of ANC4+ (<50%, 50%-75%, and 75% or more), and by regions based on the Sustainable Development Goals regional grouping.

Within our second aim, we assessed the characteristics of women with timely ANC initiation by comparing their demographic, socio-economic and health systems factors to that of women with delayed ANC initiation, stratifying by ANC4+ coverage levels. We then fit a multiple logit regression of timely ANC initiation on these factors to uncover the significant predictors.

We quantified the associations between timing of first ANC and the number as well as the content of ANC by running four different multiple regression analyses. First, we fitted a linear regression of ANC contacts on gestational age at the first ANC in months; then we ran separate logistic regressions of ANC4+ and ANC8+ on timely ANC initiation. Lastly, we ran a multinomial ordered logistic regression of the ANC content score, treated as a categorical dependent outcome, on timely ANC initiation, treated as a binary independent variable. All regression models were adjusted for country as well as demographic and socio-economic factors.

Finally, we estimated the coverage of 0, 1-3, 4-7, 8-10 ANC contacts by countries’ ANC4+ coverage levels and geographic regions, and characterized women who received 8-10 contacts compared to those who had 1-7 contacts by demographic, socio-economic and health systems variables.

All analyses were conducted with Stata 15 [20] and took into account the sampling weights and the complex survey design. Figures were generated on Microsoft Excel 2016 (Microsoft Inc, Seattle WA, USA) and Stata 15 (Stata Corp, College Station, TX, USA).

### Ethical consideration

DHS and MICS are publicly available de-identified data. Ethical approval was not needed for analysis of the data. Ethical approval for data collection was the responsibility of the institutions that collected the data.

## RESULTS

### Coverage and distribution of timely ANC initiation

**Figure 1:**
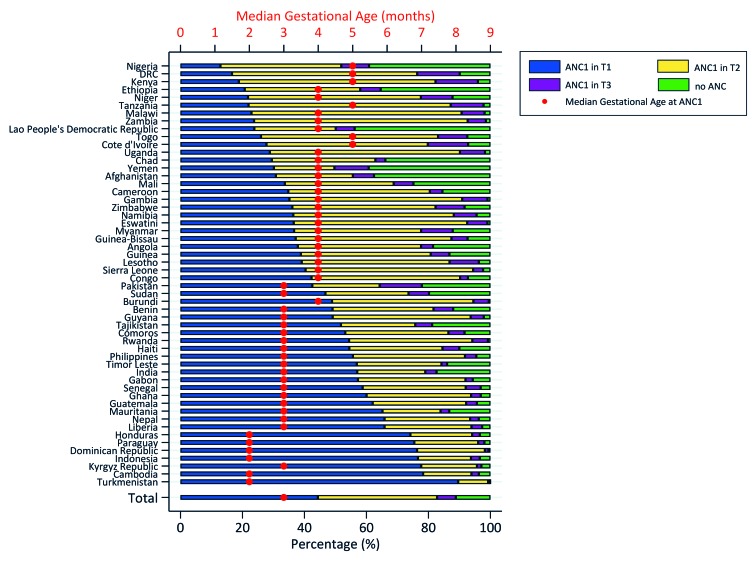
Distribution of births by gestation trimester at first antenatal care (ANC) contact, and median gestation months by country.

Across the 54 countries, the median gestational age at first ANC was three months, with an interquartile range between three and five months. Overall, 11.2% of women had no ANC during pregnancy. Half of women (49.9%) with at least one ANC contact initiated it within the first trimester of pregnancy; 43.3% and 6.8% delayed until the second and third trimesters respectively (**Table 1**). The coverage of timely ANC initiation varied by level of ANC4+ coverage and by geographic region. In countries where less than 50% of women achieved ANC4+, representing over half of the pooled data, 37.9% of women had timely ANC initiation, compared to 61.3% in countries where 75% or more women achieved ANC4+. By geographic region, Central and Southern Asia, and Latin America and the Caribbean recorded the largest proportions of timely ANC initiation among women with at least one ANC contact (69.1% and 68.1%, respectively), compared to 40.8% in Sub-Saharan Africa. **Figure 1** shows considerable disparities in timing of ANC across countries, and Figure S1 in the **Online Supplementary Document** maps out the coverage of timely ANC initiation in countries included in the analysis. However, the top 10 highest performers in terms of timely ANC initiation include countries from all regions, suggesting that achieving such performance by countries that are still behind is possible, and may not be tied to regional characteristics. Turkmenistan is the best performer in timely ANC initiation (89.6%), with almost universal use of antenatal care services. Conversely, Nigeria is the lowest performer (12.9%) with a substantial proportion of women with no ANC.

**Table 1:** Distribution of timely ANC initiation by level of ANC4+ coverage and region among women with at least one ANC*

	Median gestational age at first ANC contact (IQR)	Timing of first ANC among women with ANC1+ (N = 243 967)		
**1^st^ trimester (n = 128 495), % (95% CI)**	**2^nd^ trimester (n = 99 168), % (95% CI)**	**3^rd^ trimester (n = 16 304), % (95% CI)**
**Country groups by ANC4+ coverage level:**				
1 (<50%)	3 (3-5)	37.9 (37.2-38.7)	51.3 (50.7-52.0)	10.7 (10.3-11.1)
2 (50%-74%)	4 (3-5)	47.2 (46.5-47.9)	45.9 (45.2-46.5)	6.9 (6.6-7.2)
3 (75% +)	3 (2-4)	61.3 (60.6-62.1)	34.9 (34.2-35.6)	3.8 (3.6-4.1)
**SDG region:**				
Central and Southern Asia†	3 (2-4)	69.1 (67.9-70.3)	25.0 (24.0-26.1)	5.9 (5.4-6.4)
Eastern and South-Eastern Asia‡	3 (2-4)	63.5 (62.3-64.7)	31.4 (30.3-32.5)	5.1 (4.6-5.6)
Latin America and the Caribbean§	3 (2-4)	68.1 (66.9-69.2)	28.5 (27.4-29.7)	3.4 (3.0-3.8)
Northern Africa and Western Asia‖	3 (2-5)	54.6 (53.0-56.1)	32.9 (31.6-34.3)	12.5 (11.6-13.5)
Sub-Saharan Africa¶	4 (3-5)	40.8 (40.3-41.4)	51.7 (51.2-52.2)	7.5 (7.3-7.7)
**Total among women with ANC1+:**				
**Pooled**	**3 (3-5)**	**49.9 (49.5-50.4)**	**43.3 (42.9-43.7)**	**6.8 (6.6-7.0)**

ANC – antenatal care, IQR – interquartile range, CI – confidence interval, SDG – Sustainable Development Goal

*Note: % are row percentages, n unweighted.

†Central and Southern Asia: Afghanistan, India, Kyrgyz Republic, Nepal, Pakistan, Tajikistan, Turkmenistan.

‡Eastern and South-Eastern Asia: Cambodia, Indonesia, Lao People’s Democratic Republic, Myanmar, Philippines, Timor Leste.

§Latin America and the Caribbean: Dominican Republic, Guatemala, Guyana, Haiti, Honduras, Paraguay.

‖North Africa and Western Asia: Sudan, Yemen.

¶Sub-Saharan Africa: Angola, Burundi, Comoros, Eswatini, Ethiopia, Kenya, Lesotho, Malawi, Namibia, Rwanda, Uganda, Tanzania, Zambia, Zimbabwe, Benin, Cameroon, Chad, Congo, Cote D’Ivoire, Democratic Republic of Congo, Gabon, Gambia, Ghana, Guinea, Guinea-Bissau, Liberia, Mali, Mauritania, Niger, Nigeria, Senegal, Sierra Leone, Togo.

### Who are the women who initiate ANC on time?

Table 2 presents socio-economic and demographic characteristics of women with timely ANC initiation, as well as health systems characteristics as reported by them, stratified by the three groups of countries based on ANC4+ coverage. Overall, compared to women with delayed ANC initiation, a significantly higher proportion of women with timely ANC initiation resided in urban areas (39.5% vs 30.7%), had secondary or higher education (48.7% vs 33.2%), lived in smaller households of one to four members (28.2% vs 24.6%), and belonged to the highest wealth quintile (21.0% vs 13.1%). They were at their first child (30.4% vs 24.4%) or a child with birth order between 2 and 4 (51.7% vs 48.6%), had birth with preceding interval of five years or more (16.1% vs 13.5%), and were married (75.2% vs 71.1%). In terms of health systems characteristics, a larger proportion of women with timely ANC initiation received antenatal care in a hospital (35.5% vs 28.7%) or from the formal private sector at least once, had an institutional birth (77.4% vs 66.3%), attended the formal public sector for delivery (65.0% vs 57.4%), and had a skilled attendant at birth (82.4% vs 70.1%). Additionally, a higher proportion of women with timely ANC initiation across the three groups received at least two or more components of ANC content (88.8% vs 79.6%).

**Table 2:** Demographic, socio-economic and health systems characteristics of women with timely ANC initiation among those with at least one ANC

ANC4+ coverage level	Country Group 1 (<50% ANC4+) (n = 133 064)†		Country Group 2 (50% -74% ANC4+) (n = 61 749)†		Country Group 3 (≥75% ANC4+) (n = 49 154)†		Pooled (n = 243 967)†	
**Women’s characteristics**	**1^st^ trimester**	**2^nd^/3^rd^ trimester**	**1^st^ trimester**	**2^nd^/3^rd^ trimester**	**1^st^ trimester**	**2^nd^/3^rd^ trimester**	**1^st^ trimester**	**2^nd^/3^rd^ trimester**
**Demographic and socio-economic**								
Median age at birth (IQR)	24 (21-28)	25 (21-29)	26 (21-31)	26 (21-31)	26 (21-31)	25 (21-31)	25 (21-29)	25 (21-30)
Residence:								
Urban	33.2*	22.5	39.0*	30.7	42.8	40.3	39.5*	30.7
Rural	66.8*	77.5	61.0*	69.3	57.2	59.7	60.5*	69.3
Education:								
No education	35.9*	43.3	28.5*	31.0	11.0*	18.2	22.0*	31.2
Primary	33.1*	37.3	31.8*	39.7	26.0	27.6	29.4*	35.5
Secondary/Higher	31.0*	19.4	39.6*	29.2	63.0*	54.3	48.7*	33.2
Number of HH members:								
1-4	27.8*	24.3	26.3*	24.1	29.9*	25.5	28.2*	24.6
5-6	28.2	28.5	28.1	28.5	32.2*	28.8	29.9*	28.6
7+	43.9*	47.2	45.7	47.4	37.9*	45.7	41.9*	46.9
Sex of head of household:								
Male	87.2	86.2	76.6*	78.5	75.7*	71.0	78.3	78.9
Female	12.8	13.8	23.4*	21.5	24.3*	29.0	21.7	21.1
Wealth index:								
Q1 (poorest)	15.8*	21.8	17.9*	23.2	21.0*	26.3	18.9*	23.6
Q2	17.1*	22.3	18.7*	23.1	20.7*	22.9	19.3*	22.8
Q3	19.3*	20.9	20.2	21.6	20.8	20.4	20.3	21.1
Q4	21.4	20.2	21.3*	19.2	19.7	18.7	20.6*	19.4
Q5 (richest)	26.4*	14.8	21.9*	12.9	17.9*	11.6	21.0*	13.1
Birth order:								
1	28.9*	21.6	29.2*	24.2	32.1*	27.9	30.4*	24.4
2-4	49.2*	46.5	49.6	48.4	54.5*	51.4	51.7*	48.6
5+	21.9*	31.8	21.2*	27.4	13.5*	20.7	17.9*	27.0
Preceding birth interval:								
First child	29.0*	21.7	29.4*	24.3	32.2*	28.0	30.6*	24.5
≤2 y	15.1*	17.0	13.4	14.4	12.6*	14.2	13.4*	15.2
3-4 y	42.9*	49.4	43.6*	48.8	35.7*	41.2	39.9*	46.9
5+ years	13.0	11.9	13.5	12.5	19.6*	16.7	16.1*	13.5
Marital status:								
Single	3.1*	4.9	4.4*	7.5	7.3*	14.9	5.4*	8.7
Married	84.6*	79.0	82.4*	75.0	65.2*	56.6	75.2*	71.1
Other	12.3*	16.1	13.2*	17.5	27.5	28.5	19.4	20.1
**Health systems**								
Place of ANC:								
Hospital	37.8*	27.6	33.9*	27.4	35.4*	31.9	35.5*	28.7
Health Center	44.0	46.2	36.8*	44.5	27.4*	34.1	34.3*	42.3
Other formal	15.7*	24.4	26.3	25.4	36.1*	31.9	28.2	26.8
Other informal	2.4*	1.8	3.0	2.7	1.2*	2.0	2.1	2.2
Sector of place for ANC:								
Public formal	76.7*	84.4	85.8*	88.3	81.4*	88.4	81.8*	86.9
Private formal	20.8*	13.8	11.2*	8.9	17.3*	9.6	16.1*	10.9
Other/ informal	2.5*	1.8	3.0	2.7	1.3*	2.0	2.1	2.2
ANC content (4 components):								
None	6.1*	11.6	2.8*	4.7	1.0*	2.4	2.6*	6.3
1 component	14.1*	21.4	7.1*	12.4	7.2	8.0	8.5*	14.1
2+ components	79.8*	67.0	90.2*	82.9	91.8*	89.7	88.8*	79.6
Place of delivery:								
Health facility	73.4*	61.0	71.6*	63.9	83.8*	75.8	77.4*	66.3
Home	25.8*	37.9	26.5*	34.6	15.2*	23.1	21.3*	32.5
Other	0.9	1.1	1.9	1.5	1.0	1.0	1.3	1.2
Sector of place of delivery:								
Public formal	58.7*	52.3	62.5*	55.4	69.8*	66.0	65.0*	57.4
Private formal	14.7*	8.7	9.8	8.8	14.4*	10.0	12.8*	9.1
Other informal	26.7*	39.0	27.7*	35.8	15.8*	24.1	22.2*	33.5
Attendant at birth:								
Unskilled	23.0*	36.0	21.7*	31.8	12.0*	20.0	17.6*	29.9
Skilled	77.0*	64.0	78.3*	68.2	88.0*	80.0	82.4*	70.1

ANC – antenatal care, IQR – interquartile range, CI – confidence interval, HH – household members

*Non-overlapping 95% confidence intervals between women who initiated ANC in the first trimester and those who initiated ANC in the second or third trimester, for that characteristic.

†Note: n is unweighted.

Similar patterns were observed in each ANC4+ coverage group, with few exceptions. In the low ANC4+ coverage group, a larger proportion of women with timely ANC initiation attended ANC in the informal sector (2.4% vs 1.8%); this was not observed in the middle and high ANC4+ coverage groups. In the medium ANC4+ coverage group, there was a higher proportion of female-headed households (23.4% vs 21.5%). Both in the low and medium coverage groups, a higher proportion of women with timely ANC initiation lived in urban areas compared to those with delayed ANC initiation.

Across all three country groups, predictors of timely ANC initiation included education and wealth, married status, smaller household size and parity (Table S2 in the **Online Supplementary Document**).

### Association between timing of ANC initiation and number and content of ANC received

**Table 3** presents the results of the multiple regression models assessing the association between timing of ANC initiation and the number of contacts as well as the content of ANC received. Overall, women with timely ANC initiation are significantly more likely to achieve four and eight or more ANC contacts (aOR = 5.24 with 95% CI = 5.04-5.45 and 4.66 with 95% CI = 4.35-4.99 respectively, *P* < 0.001), and to receive higher ANC content during pregnancy (aOR = 1.66 with 95% CI = 1.59-1.73, *P* < 0.001). Furthermore, every one-month delay in ANC initiation reduces the total number of ANC contacts reached by 0.62 (β = -0.62 with 95% CI = -0.63, -0.61 and *P* < 0.001).

**Table 3:** Positive associations between timely ANC initiation and number and content of ANC received

Model	Independent variable	Dependent variable	Crude β† (95% CI)	Adjusted † (95% CI)	Crude OR† (95% CI)	Adjusted OR‡ (95% CI)
1	Gestational age (months) at first ANC contact	Number of ANC contacts	-0.66* (-0.67, -0.65)	-0.62* (-0.63, -0.61)		
2	Timely ANC (1st trimester)	ANC4+			5.82* (5.60, 6.04)	5.24* (5.04, 5.45)
3	Timely ANC (1st trimester)	ANC8+			5.32* (4.98, 5.68)	4.66* (4.35, 4.99)
4	Timely ANC (1st trimester)	ANC content			2.02* (1.94-2.11)	1.66* (1.59-1.73)

ANC – antenatal care, CI – confidence interval

*Coefficients significant at *P* < 0.001.

†Adjusted by country.

‡Adjusted by country, demographic and socio-economic variables.

**Figure 2:**
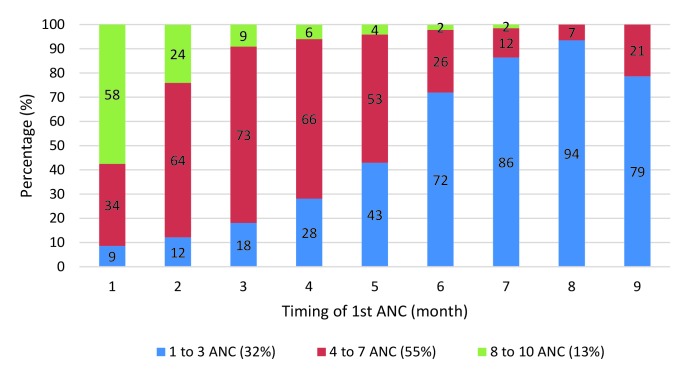
Number of antenatal care (ANC) contacts by timing of ANC initiation, among women with at least one ANC (n = 243 967).

**Figure 2** gives the breakdown of monthly gestational age at first ANC and the number of ANC contacts achieved. From the figure it can be seen that reaching eight contacts or more requires starting ANC during the first month of gestation. While 58% of women who initiated ANC in the first month achieved eight or more contacts, only 24% of those who initiated ANC in the second month achieved this number. The percentage dropped to 9% for those who initiated ANC in the third month.

Further assessment showed that over half of women who initiated ANC in the first trimester received each of the four ANC interventions (urine test, blood test, blood pressure, HIV test and received results). The proportion dropped to about 40% when ANC was initiated in the second trimester, and to 5% in the third trimester (Figure S2 in the **Online Supplementary Document**).

### Who are the women who receive a minimum of eight ANC contacts?

**Table 4** shows the distribution of the number of ANC contacts by coverage of ANC4+ and by SDG region. Overall, 11.2% of women reported not having received any ANC during pregnancy, whereas 28.3% received 1-3 contacts, 49.3% received 4-7 contacts, and 11.3% achieved eight or more contacts during pregnancy.

**Table 4:** Distribution of number of ANC contact coverage by ANC4+ country groups and regions among all women*

	Number of ANC contacts (N = 290 783)			
**No ANC** (n = 46 816)	**1-3 ANC** (n = 95 425)	**4-7 ANC** (n = 120 578)	**8-10 ANC** (n = 27 964)
**Country group by ANC4+ level:**				
	% (95% CI)	% (95% CI)	% (95% CI)	% (95% CI)
1 (<50%)	21.6 (20.9-22.4)	40.1 (39.4-40.8)	34.5 (33.8-35.2)	3.8 (3.6-4.0)
2 (50%-74%)	9.2 (8.8-9.7)	31.9 (31.3-32.5)	52.7 (52.0-53.4)	6.2 (5.8-6.5)
3 (≥75%)	4.1 (3.8-4.4)	13.9 (13.4-14.3)	58.5 (57.8-59.2)	23.5 (22.9-24.2)
**SDG regions:**				
Central and Southern Asia†	14.9 (13.9-16.0)	27.5 (26.4-28.7)	44.2 (42.9-45.6)	13.3 (12.5-14.1)
Eastern and South-Eastern Asia‡	14.0 (13.2-14.8)	17.6 (16.7-18.5)	46.8 (45.7-47.9)	21.7 (20.8-22.7)
Latin America and the Caribbean§	4.3 (3.9-4.8)	12.1 (11.3-13.0)	49.3 (48.0-50.5)	34.3 (33.0-35.7)
Northern Africa and Western Asia‖	29.6 (28.1-31.0)	32.8 (31.6-34.1)	29.9 (28.5-31.2)	7.8 (7.1-8.5)
Sub-Saharan Africa¶	9.7 (9.3-10.1)	32.3 (31.8-32.8)	51.9 (51.4-52.5)	6.1 (5.8-6.3)
**Total:**				
**Pooled**	**11.2 (10.9-11.5)**	**28.3 (27.9-28.7)**	**49.3 (48.8-49.7)**	**11.3 (11.0-11.6)**

ANC – antenatal care, IQR – interquartile range, CI – confidence interval

*Note: % are row percentages, n unweighted.

†Central and Southern Asia: Afghanistan, India, Kyrgyz Republic, Nepal, Pakistan, Tajikistan, Turkmenistan.

‡Eastern and South-Eastern Asia: Cambodia, Indonesia, Lao People’s Democratic Republic, Myanmar, Philippines, Timor Leste.

§Latin America and the Caribbean: Dominican Republic, Guatemala, Guyana, Haiti, Honduras, Paraguay.

‖North Africa and Western Asia: Sudan, Yemen.

¶Sub-Saharan Africa: Angola, Burundi, Comoros, Eswatini, Ethiopia, Kenya, Lesotho, Malawi, Namibia, Rwanda, Uganda, Tanzania, Zambia, Zimbabwe, Benin, Cameroon, Chad, Congo, Cote D’Ivoire, Democratic Republic of Congo, Gabon, Gambia, Ghana, Guinea, Guinea-Bissau, Liberia, Mali, Mauritania, Niger, Nigeria, Senegal, Sierra Leone, Togo.

Coverage of ANC8+ varies by country groups of ANC4+ coverage. ANC8+ coverage is at 3.8% in countries where ANC4+ is <50% and 6.2% when ANC4+ is between 50% and 75%; in countries with ANC4+ coverage above 75%, one in four women achieved eight or more contacts. Regionally, Latin America and the Caribbean had the highest coverage of ANC8+ at 34.3% compared to the lowest in Sub-Saharan Africa at 6.1%; it also had the highest ANC utilization, with 4.3% of women reporting no ANC (**Table 4**).

**Figure 3:**
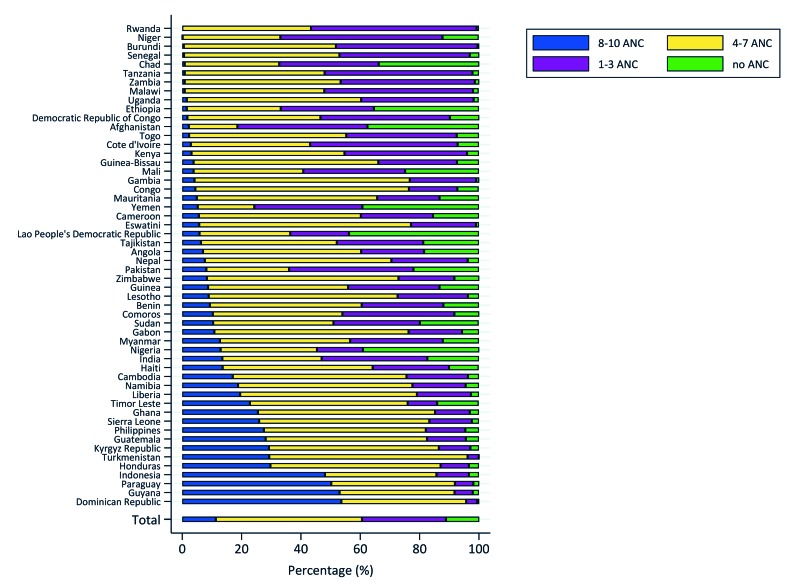
Distribution of number of antenatal care (ANC) contacts achieved by country.

**Figure 3** illustrates the distribution of the number of ANC contacts achieved by country, depicting large variations in ANC8+ ranging from 0% in Rwanda to 53.6% in the Dominican Republic.

The characteristics of women achieving a minimum of eight contacts (Table S3 in the **Online Supplementary Document**) were similar to those with timely ANC initiation. In all three country groups categorized by ANC4+ coverage levels, a significantly larger proportion of women receiving 8-10 ANC compared to 1-7 ANC were urban residents, had secondary or higher education, belonged to the top two wealth quintiles, had a parity of one, were married, received ANC from a hospital or in the private formal sector, and received at least two components of ANC content. Additionally, a larger proportion of these women had an institutional delivery and a skilled attendant at birth. Conversely, in the low ANC4+ coverage group, a higher proportion of women achieving ANC8+ attended ANC in the informal sector; this was not observed in the medium and high ANC4+ coverage groups. Predictors of ANC8+ were also similar to those of timely ANC initiation (Table S4 in the **Online Supplementary Document**).

## DISCUSSION

Antenatal care coverage is an important indicator that has been globally reported on to assess maternal health. Although the evidence on number of ANC contacts is readily available, countries’ performance in terms of timing of ANC initiation is reported less frequently. Effective promotion of antenatal care and a positive pregnancy experience among women in low- and middle income countries (LMIC) requires a good understanding of the timing and patterns of ANC contacts as well as the characteristics of women who are likely to fall behind. To our knowledge, this has not been previously studied at a global scale. Our findings not only fill an important gap in the scientific literature, but serve as a call for action for decision-makers. Our analysis of 290 783 births from 54 LMIC found that less than half of the women initiated ANC within the first trimester of pregnancy as recommended by WHO. A total of 11.2% reached the newly set WHO recommendation of a minimum of 8 contacts, while 60.6% achieved at least four contacts. The coverage of timely ANC initiation varied by countries’ coverage level of ANC4+: in high coverage countries (≥75% ANC4+), 61.3% of women with at least one ANC had a timely ANC initiation compared to 37.9% in countries with low coverage (<50% ANC4+). Overall, Turkmenistan had the highest coverage of timely ANC initiation (89.6% of all women) and highest ANC utilization, and Nigeria depicted the poorest performance in timely ANC initiation (12.9% of all women). The top 10 countries with timely ANC initiation are scattered throughout three continents (Africa, Asia, America), suggesting that their performance is achievable by other countries. These are also countries that have made progress in reducing inequalities in coverage. For example, Turkmenistan has achieved almost inexistent inequalities, and is the Countdown to 2030 priority country with the highest composite coverage index (CCI), indicating high coverage of reproductive maternal and newborn health services across the continuum of care; whereas Nigeria has recorded some of the largest socio-economic inequalities and lowest CCI [5]. Regionally, Central and Southern Asia followed by Latin America and the Caribbean had the best performance of timely ANC initiation. Conversely, Sub-Saharan Africa is falling behind.

The achievement of 8-10 ANC contacts followed a similar pattern, with a quarter of women in high ANC4+ coverage countries receiving at least eight contacts, compared to 3.8% in low ANC4+ countries. This suggests that for countries where coverage of ANC4+ is below 75%, the large majority among countries in this study, achieving high coverage levels of ANC8+ will be a very steep climb. This is less so for countries with ANC4+ above 75%. Latin America and the Caribbean outperformed the other regions, with the Dominican Republic having the overall highest proportion of women reaching 8-10 ANC (53.6%), compared to Sub-Saharan Africa where Rwanda was the lowest performer with no women reaching eight contacts. Further analyses are needed to understand the specific case of Rwanda, where health systems reforms have led to improvement of maternal health outcomes [21]; however our data suggested that despite having 54.4% of women with timely initiation, none reported achieving eight contacts. The previous recommendation of ANC4+, which was adopted as a policy in all countries and implemented in routine ANC may have affected the overall report of ANC contacts by pregnant women. A cross-sectional study assessing factors of poor ANC utilization in Rwanda indicated that older age, single status, large households and lack of social support were associated with poor ANC utilization of two or less contacts during pregnancy [22].

Other studies have also documented the high ANC performance in Latin America and Caribbean [23]: although this region has the largest income inequalities globally, with varying ANC performance across countries, programs targeting the most vulnerable such as performance-based contracts and conditional-cash transfer schemes have been successful at promoting ANC utilization in recent years [23,24].

Our analyses suggested a strong, independent effect of timing of ANC initiation on the number of contacts, as well as the content of care received. Women with timely ANC initiation had 5.2 (95% CI = 5.0-5.5) and 4.7 (95% CI = 4.4-5.0) times higher odds of achieving at least four and eight ANC contacts respectively, and 1.7 (95% CI = 1.6-1.7) times higher odds of receiving a higher ANC content overall. Further analysis by month of gestation showed that even among women who initiated ANC in a timely manner, those who started in the first month were more likely to report a minimum of 8 contacts. It is unclear whether the ANC contact schedule of these women was consistent with the recommended schedule by WHO, given that DHS and MICS surveys only capture the timing of the first contact.

Women with low education and wealth status, living in larger households, having short birth intervals and higher parity were less likely to begin antenatal care in a timely manner and achieve eight contacts. Strategies to increase ANC contacts must target these women. Our findings were consistent with other studies [1,24-28]: one of them examined coverage and timing of ANC among the poor in Mesoamerican countries and showed that education, parity and marital status were factors predicting timely ANC initiation [24]. Similarly, Gupta et al. looked at utilization of antenatal care in Tanzania between 1990 and 2010 and found that urban residence, lower birth order and ANC initiation before four months of gestation were associated with utilization of at least four antenatal care visits [1].

A number of studies have explored reasons for no or delayed ANC initiation, and have included factors such as financial constraints, distance from the health facility, lack of knowledge about the recommended timing of ANC initiation, and socio-cultural factors such as lack of permission from spouse, late disclosure of pregnancy status, pregnancy wantedness [12-16,18,19,29,30]. These factors, in addition to the characteristics studied here of women falling behind in terms of timing and number of ANC, should be taken into consideration for specific ANC targeting and programmatic purposes.

The newly set WHO recommendation of a minimum of eight ANC contacts, with higher frequency of contacts almost every two weeks in the third trimester, did not identify effective strategies to overcome the challenges and obstacles mentioned above. Our analyses suggest that achieving such target will be a steep climb for most countries, who will need to put in place policy and programs that tackle both service demand and the strengthening of the health system, including the delivery mechanisms of services, infrastructures and commodities, and the monitoring of these services. It will not be surprising if these countries are hesitant to adopt the new recommendations, or worse, implement it only in areas that already have higher ANC coverage, thus further increasing equity gaps. Supportive programmatic measures and resources will be needed to avoid disturbance to already fragile systems. Implementation research can help identify effective and scalable strategies.

Quality of antenatal care content is difficult to measure in household surveys, given that existing questions in these surveys are limited and focus on receipt of components reported by the mother, and don’t assess the quality per say of services received. Benova and colleagues examined the coverage and content of ANC in 10 LMIC, and indicated the need for improved measurement of ANC quality: their results suggested that content of care was poor even among women with adequate number and timing of ANC [31]. Our study suggested that women initiating ANC in a timely manner were more likely to receive a higher number of preventive screening components as part of ANC. Mixed methods and qualitative studies are crucial to get a better picture of the quality of services received, including respectful care and satisfaction. Furthermore, linking household surveys to health facility assessments will also be an important step towards improved coverage measurement.

Our analysis has several limitations. The variables on timing, number and content of antenatal care were based on self-reported events, which are subject to recall-bias. This was also the case for the assessment of gestational age based on self-report by the mother. Limiting the analysis to the last live birth in the two years preceding the survey may have reduced this bias. Coverage estimates rely heavily on self-reported data obtained from household surveys, yet little is known on the validity of these estimates, particularly in LMIC settings. A validation study in Southwestern China in 2011 found that self-reported coverage of routine ANC interventions had overall poor validity, and timing of 1st ANC prior to 12 weeks of gestation had a large population-level bias [32]. In our study, the recall of timing of first ANC was recorded in months rather than weeks, and there could have been a bias leading to overestimation of the proportion of women receiving ANC1 in the first trimester. In addition, there may have been social desirability bias in the number of ANC reported, although this was less likely to be an issue for ANC8+ given it has not yet been adopted widely. We excluded women who reported more than ten contacts; although we considered these to be over-reporting cases, they could also describe ANC behaviors for very sick mothers or those with high obstetric risks. However, the proportion was low (3.8% of the pooled data set) and would have negligible effects on our results. We assessed content of ANC using four components reported to have been received at least once during ANC. These were not comprehensive nor the most important components of antenatal care, but rather the ones most commonly reported in DHS and MICS surveys. Moreover, no information was available on the contacts at which these components were received.

## CONCLUSIONS

In conclusion, our findings from 54 priority Countdown countries show that timely ANC initiation is a major driving force for meeting the 2016 WHO antenatal care guidelines for a positive pregnancy experience. This achievement appears feasible in short to medium term in countries where coverage of ANC4+ is very high. For most countries, achieving high coverage levels of ANC8+ will require strong policy change and implementation, and dedicated resources to reaching vulnerable and poor populations that are still not accessing the services. In order to ensure that no woman is left behind, programs and policies promoting antenatal care services ought to bridge the gap and focus on the most vulnerable women whose needs in antenatal care aren’t being met. Strong implementation research is needed to learn about effective strategies.

## Additional material

Online Supplementary Document
