# Supplementary material for: Timing and number of antenatal care contacts in low and middle-income countries: Analysis in the Countdown to 2030 priority countries
Source: J Glob Health. 2020 Feb 20;10(1):010502. doi: 10.7189/jogh.10.010502 (PMC7101027; doi:10.7189/jogh.10.010502)
Supplement: Online Supplementary Document [file jogh-10-010502-s001.pdf]

Timing and Number of Antenatal Care Contacts in Low and Middle-Income Countries: Analysis in the Countdown to 2030 Priority Countries

[Antenatal care in Countdown to 2030 priority Countries: Timing Matters]

<u>Online Supplementary Document</u>

Table S1: Distribution of timing of ANC initiation and median gestational age at first ANC among all women with 0-10 ANC in countries with available data.

Country	Data Source	Last birth in 2 years preceding survey * (n)	ANC4+ coverage (%)	Country group based on ANC4+ coverage	Median Gestational Age at first ANC visit (months)	Timing of ANC initiation (%)			
						1st Trimester	2nd Trimester	3rd Trimester	No ANC
Afghanistan	DHS 2015	11,365	18.7	1	4	30.8	24.8	6.8	37.6
Angola	DHS 2015-16	5,653	60.3	2	4	37.9	39.8	3.9	18.5
Benin	DHS 2011-12	4,813	60.5	2	3	49.0	32.6	6.3	12.0
Burundi	DHS 2016-17	5,191	51.8	2	4	48.9	45.6	4.9	0.6
Cambodia	DHS 2014	2,871	75.5	3	2	78.3	15.6	2.4	3.7
Cameroon	MICS 2014	2,738	60.2	2	4	34.8	45.7	4.0	15.5
Chad	DHS 2014-2015	6,459	32.6	1	4	29.5	33.4	3.2	33.9
Comoros	DHS 2012	1,053	54.0	2	3	53.2	33.3	5.2	8.4
Congo	DHS 2011-12	3,811	76.4	3	4	42.3	48.0	2.5	7.2
Côte d'Ivoire	DHS 2011-2012	3,073	43.1	1	5	27.7	52.1	13.0	7.2
Democratic Republic of the Congo	DHS 2013-2014	7,213	46.5	1	5	16.6	59.8	13.7	9.9
Dominican Republic	DHS 2013	1,036	95.7	3	2	76.3	21.9	1.1	0.7
Eswatini	MICS 2014	935	77.1	3	4	36.5	56.0	6.5	1.0
Ethiopia	DHS 2016	3,982	33.2	1	4	20.7	37.3	6.6	35.4
Gabon	DHS 2012	2,473	76.3	3	3	57.3	34.7	2.3	5.7
Gambia	DHS 2013	3,465	76.7	3	4	35.1	55.8	8.1	1.0

Country	Data Source	Last birth in 2 years preceding survey * (n)	ANC4+ coverage (%)	Country group based on ANC4+ coverage	Median Gestational Age at first ANC visit (months)	Timing of ANC initiation (%)			
						1st Trimester	2nd Trimester	3rd Trimester	No ANC
Ghana	DHS 2014	2,225	85.2	3	3	60.0	33.7	3.1	3.1
Guatemala	DHS 2014-2015	4,197	82.6	3	3	62.0	30.2	3.5	4.4
Guinea	DHS 2012	2,775	56.0	2	4	38.8	41.9	6.0	13.3
Guinea-Bissau	MICS5 2014	2,959	66.1	2	4	37.1	50.3	5.2	7.4
Guyana	MICS5 2014	932	91.8	3	3	49.1	44.5	4.2	2.1
Haiti	DHS 2012	2,844	64.3	2	3	54.5	30.1	5.4	10.0
Honduras	DHS 2011-2012	4,324	87.1	3	2	74.2	20.0	2.5	3.4
India	DHS 2015-16	91,614	47.0	1	3	57.0	22.0	3.6	17.4
Indonesia	DHS 2012	6,165	85.7	3	2	76.6	17.1	2.8	3.4
Kenya	DHS 2014	7,898	54.7	2	5	18.8	63.5	13.7	4.0
Kyrgyz Republic	DHS 2012	1,662	86.4	3	3	77.7	18.0	1.4	2.9
Lao People's Democratic Republic	MICS 2011-2012	4,218	36.4	1	4	23.8	26.3	6.1	43.8
Lesotho	DHS 2014	1,354	72.6	2	4	39.1	47.7	9.4	3.7
Liberia	DHS 2013	2,810	79.1	3	3	65.8	28.1	3.4	2.7
Malawi	DHS 2015-16	6,474	48.0	1	4	22.9	67.8	7.4	1.9
Mali	DHS 2012-13	3,867	40.9	1	4	33.7	35.1	6.3	24.9
Mauritania	MICS 2015	3,848	65.7	2	3	65.2	18.8	2.8	13.3
Myanmar	DHS 2015-16	1,742	56.6	2	4	36.7	41.0	10.2	12.2
Namibia	DHS 2013	1,534	77.6	3	4	36.4	51.8	7.3	4.5
Nepal	DHS 2016	1,903	70.5	2	3	65.8	27.6	2.8	3.8
Niger	DHS 2012	4,722	33.2	1	4	21.8	55.8	10.2	12.2
Nigeria	DHS 2013	10,462	45.4	1	5	12.9	39.0	8.9	39.2
Pakistan	DHS 2012-13	3,995	36.0	1	3	42.5	21.8	13.6	22.1
Paraguay	MICS 2016	1,198	91.9	3	2	75.4	20.6	2.0	2.0

Country	Data Source	Last birth in 2 years preceding survey * (n)	ANC4+ coverage (%)	Country group based on ANC4+ coverage	Median Gestational Age at first ANC visit (months)	Timing of ANC initiation (%)			
						1st Trimester	2nd Trimester	3rd Trimester	No ANC
Philippines	DHS 2013	2,521	82.1	3	3	55.6	36.1	3.7	4.6
Rwanda	DHS 2014-2015	3,166	43.4	1	3	54.4	39.8	5.0	0.8
Senegal	DHS_2016	2,559	53.0	2	3	58.8	33.3	4.8	3.2
Sierra Leone	DHS 2013	3,258	83.4	3	4	40.3	54.1	3.2	2.4
Sudan	MICS 2014	5,316	51.0	2	3	46.7	26.9	6.6	19.8
Tajikistan	DHS 2012	1,889	52.1	2	3	51.8	24.0	5.4	18.8
Tanzania	DHS 2015-2016	4,119	48.0	1	5	21.9	65.3	10.6	2.2
Timor Leste	DHS 2016	2,772	76.1	3	3	56.9	27.3	1.8	14.0
Togo	DHS 2013-2014	2,766	55.4	2	5	26.0	57.1	9.4	7.5
Turkmenistan	MICS 2015-2016	1,301	96.2	3	2	89.6	9.7	0.6	0.1
Uganda	DHS 2016	5,890	60.3	2	4	28.8	61.3	8.1	1.7
Yemen	DHS 2013	6,079	24.3	1	4	30.1	19.5	11.0	39.3
Zambia	DHS 2013-2014	5,040	53.3	2	4	23.7	68.9	5.9	1.5
Zimbabwe	DHS 2015	2,254	72.9	2	4	36.1	46.3	9.3	8.4
**Total**	-	**290,783**	**60.6**	-	**3**	**44.3**	**38.5**	**6.0**	**11.2**

*n unweighted and restricted to last birth 2 years preceding survey with 0-10 reported ANC contacts

Figure S1: Map of timely ANC initiation coverage in countries included in the analysis.

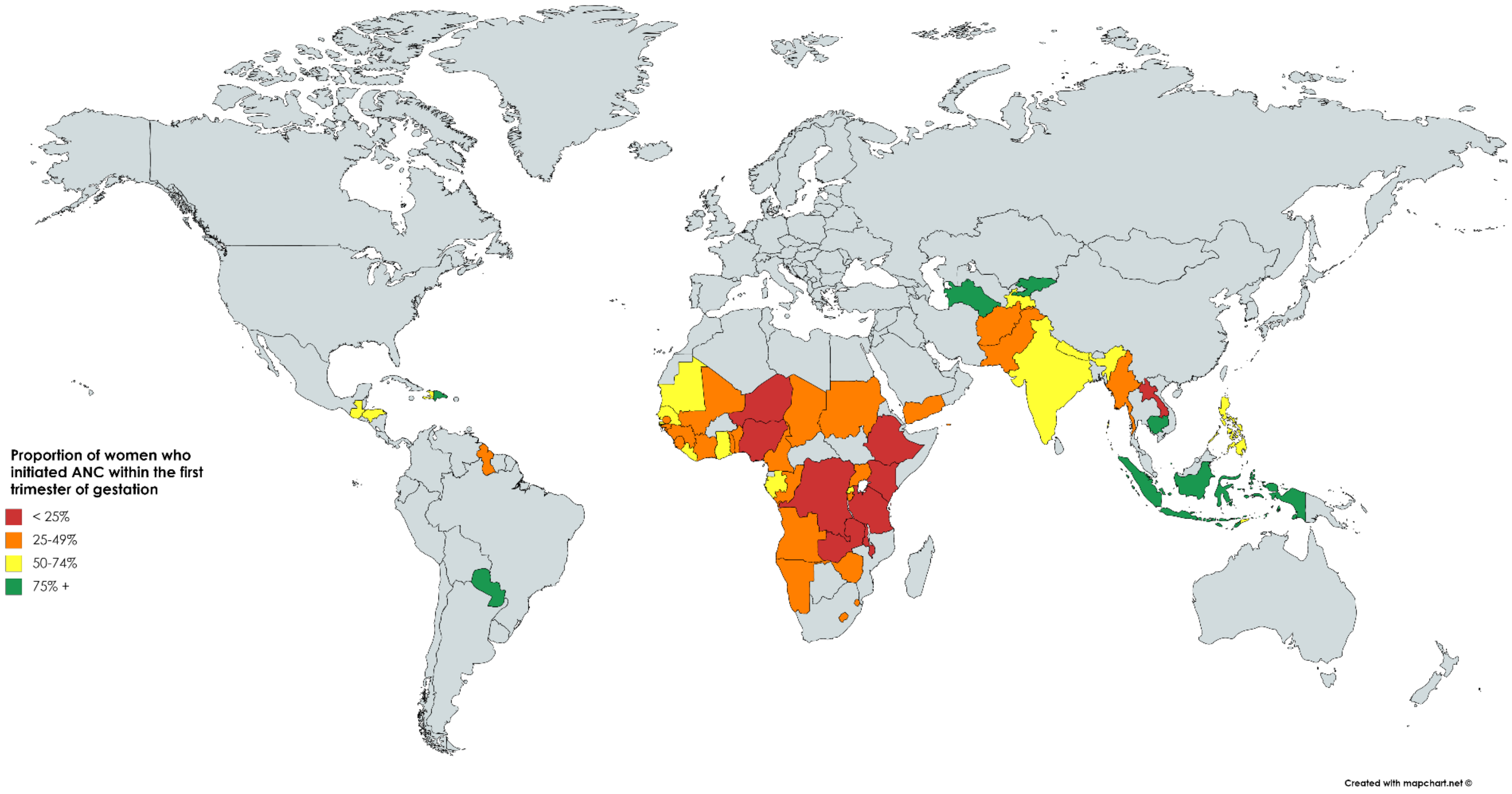

Table S2: Predictors of timely ANC initiation by ANC4+ country groups among women with at least one ANC contact.

Women's characteristics		Country Group 1 (<50% ANC4+)	Country Group 2 (50%-74% ANC4+)	Country Group 3 (≥75% ANC4+)
		aOR	aOR	aOR
**Demographic and Socio-Economic**				
Mother's age at birth	(years)	**1.01***	**1.01***	**1.02***
Residence	Urban (ref)			
	Rural	**0.85***	1.05	**1.25***
Education	No education (ref)			
	Primary	**1.19***	**1.16***	**1.11***
	Secondary	**1.33***	**1.32***	**1.22 ***
	Higher	**2.23***	**2.13***	**1.90***
Number of HH members	1-4 (ref)			
	5-6	**0.88***	**0.88***	**0.89***
	7+	**0.83***	**0.81***	**0.83***
Marital status	Single (ref)			
	Married	**1.73***	**1.63***	**1.70***
	Other	**1.46***	**1.35***	**1.32***
Sex of head of household	Male (ref)			
	Female	**1.08***	1.01	0.98
Wealth Index	Q1 (poorest) (ref)			
	Q2	1.04	0.98	**1.14***
	Q3	**1.21***	**1.10***	**1.26***
	Q4	**1.30***	**1.25***	**1.33***
	Q5 (richest)	**1.81***	**1.86***	**1.86***
Birth order	1 (ref)			
	2-4	**0.89***	0.92	0.91
	5+	**0.73***	**0.75***	**0.68***
Preceding Birth Interval	First child (ref)			
	≤2 years	**0.81***	**0.80***	**0.73***
	3-4 years	0.92	**0.86***	0.93
	5+ years	-	-	-

* significant at P<0.05

Figure S2: ANC content received by timing of ANC initiation.

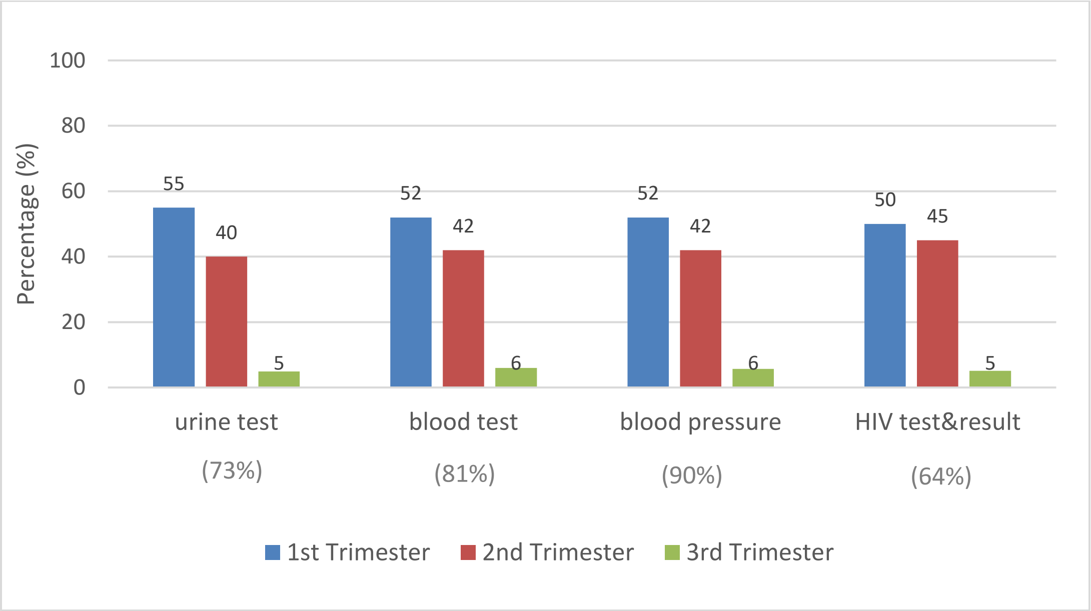

Table S3: Distribution of demographic, socio-economic and health systems characteristics of women achieving 8-10 ANC among women with at least one ANC contact.

ANC4+ coverage level		Country Group 1<br><50% ANC4+<br>(n=133,064)		Country Group 2<br>50%-74% ANC4+<br>(n=61,749)		Country Group 3<br>≥75% ANC4+<br>(n=49,154)		Pooled<br>(n=243,967)	
**Women's characteristics**		**1-7 ANC**	**8-10 ANC**	**1-7 ANC**	**8-10 ANC**	**1-7 ANC**	**8-10 ANC**	**1-7 ANC**	**8-10 ANC**
**Demographic and Socio-Economic**									
Median age at birth	Median (IQR)	24 (21-29)	25 (22-28)	26 (21-31)	26 (22-31)	25 (21-31)	26 (22-31)	25 (21-30)	25 (22-30)
Residence	Urban	25.2	**53.0***	33.2	**54.0***	39.0	**50.3***	32.8	**51.3***
	Rural	74.8	**47.0***	66.8	**46.0***	61.0	**49.7***	67.2	**48.7***
Education	No education	41.5	**21.3***	30.7	**18.3***	15.5	**8.6***	28.8	**11.7***
	Primary	36.5	**18.9***	36.7	**27.0***	27.9	**22.8***	33.8	**23.3***
	Secondary/Higher	21.9	**59.8***	32.7	**54.6***	56.7	**68.5***	37.4	**65.0***
Number of HH members	1-4	25.5	27.6	24.9	**28.5***	26.7	**32.8***	25.7	**31.4***
	5-6	28.3	29.8	28.2	28.6	30.1	**33.2***	28.9	**32.0***
	7+	46.1	**42.6***	46.9	**42.8***	43.2	**34.0***	45.5	**36.6***
Sex of head of household	Male	86.5	87.9	77.8	**74.7***	73.6	74.6	79.0	**76.0***
	Female	13.5	12.1	22.2	**25.3***	26.4	25.4	21.0	**24.0***
Wealth Index	Q1 (poorest)	20.2	**5.9***	21.5	**9.6***	25.4	**15.9***	22.4	**13.6***
	Q2	20.8	**11.7***	21.7	**11.9***	22.6	**18.5***	21.7	**16.5***
	Q3	20.4	**17.6***	21.2	**17.2***	20.8	20.1	20.9	**19.3***
	Q4	20.5	**23.9***	19.8	**25.2***	18.2	**22.5***	19.5	**23.2***
	Q5 (richest)	18.1	**40.8***	15.8	**36.1***	13.0	**23.1***	15.5	**27.5***
Birth order	1	23.8	**34.9***	26.0	**34.6***	29.2	**34.2***	26.4	**34.4***
	2-4	47.4	49.6	48.9	49.4	52.9	54.4	49.8	**52.9***
	5+	28.7	**15.4***	25.1	**16.0***	17.9	**11.3***	23.9	**12.7***
Preceding Birth Interval	First child	23.9	**35.1***	26.1	**35.0***	29.3	**34.5***	26.5	**34.6***
	≤2 years	16.4	14.4	14.2	**10.2***	13.8	**11.4***	14.7	**11.4***
	3-4 years	47.5	**36.7***	47.1	**36.7***	39.5	**32.5***	44.8	**33.8***
	5+ years	12.2	13.8	12.6	**18.0***	17.4	**21.6***	14.0	**20.1***
Marital status	Single	4.3	**1.8***	6.2	**4.8***	11.4	**6.7***	7.3	**5.8***
	Married	80.5	**93.7***	78.2	**83.0***	60.5	**66.1***	73.3	72.2
	Other	15.2	**4.6***	15.7	**12.3***	28.1	27.2	19.4	**22.0***

Health Systems									
Place of ANC	Hospital	29.9	**64.9***	28.9	**51.1***	31.8	**41.1***	30.1	**45.7***
	Health Center	46.5	**22.2***	42.0	**25.1***	33.2	**19.8***	40.7	**21.1***
	Other Formal	21.7	**9.9***	26.1	**21.5***	33.4	**37.7***	27.0	**31.5***
	Other Informal	1.9	**3.0***	2.9	2.3	1.6	1.5	2.2	1.8
Sector of place for ANC	Public formal	82.6	**58.7***	87.7	**79.4***	87.2	**74.3***	85.9	**73.5***
	Private formal	15.4	**37.9***	9.4	**18.3***	11.2	**24.2***	11.9	**24.6***
	Other/ informal	2.0	**3.3***	2.9	2.3	1.6	1.5	2.2	1.9
ANC content (4 components)	None	9.9	**2.1***	4.0	**1.4***	1.8	**0.9***	5.0	**1.1***
	1 component	19.3	**6.6***	10.2	**4.5***	7.8	**6.6***	12.1	**6.2***
	2+ components	70.8	**91.3***	85.8	**94.1***	90.4	**92.5***	83.0	**92.7***
Place of delivery	Health Facility	64.7	**84.4***	66.7	**78.8***	78.8	**86.7***	69.9	**84.9***
	Home	34.2	**15.3***	31.6	**20.0***	20.2	**12.4***	28.7	**14.2***
	Other	1.1	**0.3***	1.7	1.3	1.1	0.8	1.3	**0.9***
Sector of Place of Delivery	Public formal	54.8	52.4	58.5	**62.8***	68.2	68.5	60.5	**65.7***
	Private formal	9.9	**31.8***	8.8	**16.5***	10.8	**18.7***	9.7	**19.6***
	Other/ informal	35.3	**15.8***	32.8	**20.7***	21.0	**12.8***	29.8	**14.7***
Skilled attendant at birth	Unskilled	32.0	**12.9***	28.0	**14.1***	17.0	**9.3***	25.7	**10.6***
	Skilled	68.0	**87.1***	72.0	**85.9***	83.0	**90.7***	74.3	**89.4***

Note: n unweighted

**Non-overlapping 95% confidence intervals between women who had 1-7 ANC contacts and those who had 8-10 ANC contacts, for that characteristic.*

Table S4: Predictors of ANC8+ by ANC4+ country groups among women with at least one ANC contact

Women's characteristics		Country Group 1 (<50% ANC4+)	Country Group 2 (50%-74% ANC4+)	Country Group 3 (≥75% ANC4+)
		aOR	aOR	aOR
**Demographic and Socio-Economic**				
Mother's age at birth	(years)	**1.03***	**1.02***	**1.02***
Residence	Urban (ref)			
	Rural	**0.74***	**0.80***	0.96
Education	No education (ref)			
	Primary	**1.40***	**1.30***	1.05
	Secondary	**2.02***	**1.56***	**1.16***
	Higher	**2.61***	**2.58***	**1.80***
Number of HH members	1-4 (ref)			
	5-6	**0.85***	0.93	0.92
	7+	**0.73***	0.99	**0.87***
Marital status	Single (ref)			
	Married	**1.48***	**1.52***	**1.37***
	Other	1.03	1.26	**1.21***
Sex of head of household	Male (ref)			
	Female	**1.16***	**1.14***	1.04
Wealth Index	Q1 (poorest) (ref)			
	Q2	**1.54***	1.11	**1.33***
	Q3	**1.95***	**1.48***	**1.58***
	Q4	**2.20***	**1.97***	**2.05***
	Q5 (richest)	**3.66***	**3.01***	**2.87***
Birth order	1 (ref)			
	2-4	0.85	0.99	0.94
	5+	**0.64***	**0.77***	**0.77***
Preceding Birth Interval	First child (ref)			
	≤2 years	0.82	**0.68***	**0.78***
	3-4 years	0.88	**0.78***	0.93
	5+ years	-	-	-

* significant at P<0.05
